# IGF2BP3-mediated m^6^A modification of RASGRF1 promoting joint injury in rheumatoid arthritis

**DOI:** 10.1038/s41413-025-00434-z

**Published:** 2025-05-12

**Authors:** Qishun Geng, Yi Jiao, Wenya Diao, Jiahe Xu, Zhaoran Wang, Xing Wang, Zihan Wang, Lu Zhao, Lei Yang, Yilin Wang, Tingting Deng, Bailiang Wang, Cheng Xiao

**Affiliations:** 1 China-Japan Friendship Clinical Medical College, Chinese Academy of Medical Sciences & Peking Union Medical College, Beijing, China; 2 Institute of Clinical Medical Sciences, China-Japan Friendship Hospital, Beijing, China; 3 Beijing University of Chinese Medicine, China-Japan Friendship Hospital Clinical Medicine, Beijing, China; 4 Peking University China-Japan Friendship School of Clinical Medicine, Beijing, China; 5 Department of TCM Rheumatology, China-Japan Friendship Hospital, Beijing, China; 6 China-Japan Friendship Hospital, Capital Medical University, Beijing, China; 7 Department of Pathology, China-Japan Friendship Hospital, Beijing, China; 8 Beijing Friendship Hospital, Capital Medical University, Beijing, China; 9 Department of Orthopaedic Surgery, China-Japan Friendship Hospital, Beijing, China; 10 Department of Emergency, China-Japan Friendship Hospital, Beijing, China

**Keywords:** Pathogenesis, Bone

## Abstract

With the deepening of epigenetic research, studies have shown that N^6^-methyladenosine (m^6^A) is closely related to the development of rheumatoid arthritis (RA), but the mechanism is still unclear. In the study, we collected synovial tissues from normal controls and patients with osteoarthritis (OA) or RA. The levels of m^6^A and inflammation were analyzed by immunofluorescence staining and western blotting. The roles of IGF2BP3 in cell proliferation and inflammatory activation were explored using transfection and RNA immunoprecipitation assays. *IGF2BP3*^*−/−*^ mice were generated and used to establish an arthritis mouse model by transferring serum from adult arthritis K/BxN mice. We found m^6^A levels were markedly increased in RA patients and mouse models, and the expression of IGF2BP3 was upregulated in individuals with RA and related to the levels of inflammatory markers. IGF2BP3 played an important part in RA-fibroblast-like synoviocytes (FLS) by promoting cell proliferation, migration, invasion, inflammatory cytokine release and inhibiting autophagy. In addition, IGF2BP3 inhibited autophagy to reduce ROS production, thereby decreasing the inflammatory activation of macrophages. More importantly, RASGRF1-mediated mTORC1 activation played a crucial role in the ability of IGF2BP3 to promote cell proliferation and inflammatory activation. In an arthritis model of *IGF2BP3*^*−/−*^ mice, IGF2BP3 knockout inhibited RA-FLS proliferation and inflammatory infiltration, and further ameliorated RA joint injury. Our study revealed an important role for IGF2BP3 in RA progression. The targeted inhibition of IGF2BP3 reduced cell proliferation and inflammatory activation and limited RA development, providing a potential strategy for RA therapy.

## Introduction

Rheumatoid arthritis (RA) is a chronic and systemic disease mainly characterized by inflammatory synovitis, whose etiology is unknown.^1^ RA is often accompanied by the involvement of extra-articular organs and positive serum rheumatoid factors, which can lead to joint deformity and loss of joint function.^2^ RA-associated joint destruction is largely attributed to fibroblast-like synoviocytes (FLS) -mediated proliferation and macrophage-mediated inflammation. During the onset of RA, the number of intraarticular FLS increases and the invasion of RA-FLS is enhanced, resulting in the transformation of the synovial lining into an invasive tissue mass.^3^ In addition, RA-FLS can serve as important immunomodulators in pathogenesis by secreting inflammatory factors (such as interleukin (IL)-6) and interacting with immune cells. FLS lead to the inflow, proliferation and survival of macrophages through the production of a series of cytokines, chemokines and proangiogenic factors.^4^ In RA patients, M1 activation promotes the production of many proinflammatory cytokines, chemokines, and matrix metalloproteinases (MMPs), leading to osteoclast formation, erosion, and progressive joint destruction.^5^ To summarize, FLS and macrophages are essential for the initiation of arthritis, and synovial cell proliferation and macrophage inflammatory activation are typical features of RA progression. Interestingly, the RAS pathway is a typical carcinogenic pathway, whose activation is also closely related to the increased adhesion, migration, proliferation and secretion of inflammatory factors by RA-FLS, and participates in the polarization of macrophages.^6,7^ However, the regulatory mechanism of the RAS signaling pathway in patients with RA has rarely been reported.

Epigenetic mechanisms play key roles in coordinating the invasive phenotype of RA-FLS and macrophage inflammatory activation.^8,9^ In recent years, an improved understanding of epigenetic modifications has provided new perspectives for regulating biological functions at the transcriptional and posttranscriptional levels.^10^ Notably, N^6^-methyladenosine (m^6^A) is the most common, abundant and conserved form of internal mRNA modification in eukaryotic cells.^11^ Previous studies have shown that abnormal m^6^A modifications mediated by m^6^A regulators are closely related to a variety of diseases, such as heart failure, liver disease and cancer.^12^ Several studies also have reported abnormal m^6^A modifications in autoimmune diseases, including RA.^13^ Compared with those in the control group, the mRNA expression levels of ALKBH5, FTO and YTHDF2 in the peripheral blood of RA patients are significantly reduced.^14^ A large-scale genome-wide association study revealed that 37 m^6^A -SNPs were associated with RA.^15^ Notably, METTL3 has been reported to promote RA-FLS activation and inflammatory responses by activating the NF-κB signaling pathway.^16^ In addition, the ALKBH5 level in the joint synovial tissue of RA patients is higher than that in the healthy control group. Moreover, the arthritis severity of CIA rats injected with ALKBH5-shRNA was improved.^17,18^ Studies have also shown that METTL3-mediated m^6^A modification of ATG7 regulates the autophagy-GATA4 axis to promote degenerative changes in synovialis, degradation of articular cartilage matrix and secretion of inflammatory factors.^19^ At present, comprehensive data on the role of m^6^A -modified proteins in synovial proliferation and joint destruction in RA are lacking, and further studies are needed to clarify their exact roles in RA.

In this study, we investigated the regulatory effects of IGF2BP3 and m^6^A modifications on the proliferation and invasion of RA-FLS and the M1 polarization of macrophages. Interestingly, IGF2BP3 improved the stability of Ras protein-specific guanine nucleotide releasing factor 1 (RASGRF1) mRNA to activate the RAS pathway, which activated mTORC1 to promote synovial cell proliferation and inflammatory activation, thereby aggravating joint injury in individuals with RA. Thus, our study broadens the current molecular understanding of the regulatory role of RNA methylation in RA. Moreover, these findings may provide a new target for the treatment of RA.

## Results

### IGF2BP3 expression is upregulated in individuals with RA and is related to the levels of inflammatory markers

**Fig. 1:**
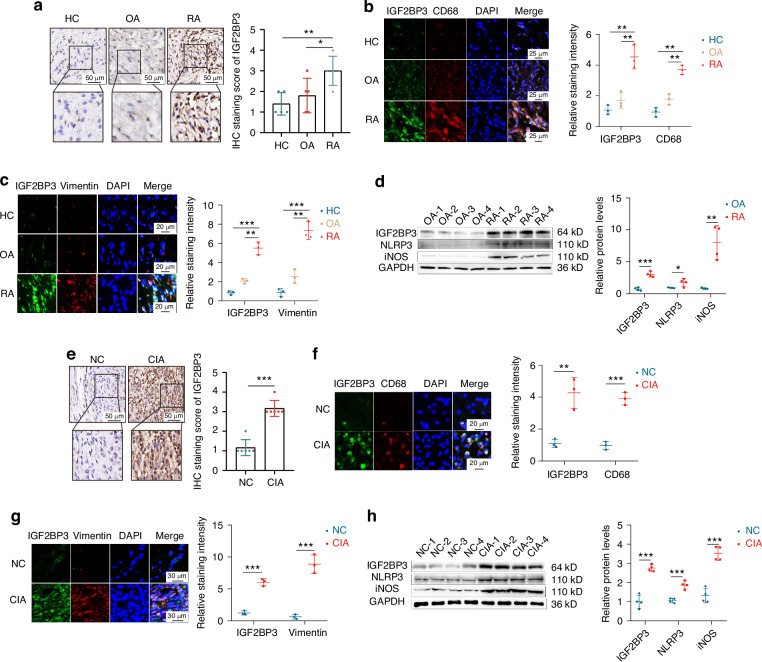
IGF2BP3 expression is upregulated and closely associated with the levels of inflammatory markers in RA. **a** IGF2BP3 expression in the synovium from healthy control (HC), OA and RA patients was detected and assessed by immunohistochemistry. **b**, **c** Immunofluorescence staining for CD68, vimentin, IGF2BP3 and DAPI in the synovium from HC, OA and RA. **d** Western blot analysis of IGF2BP3, iNOS and NLRP3 protein levels in the synovium from RA and OA. **e** The expression of IGF2BP3 in the synovium of normal control (NC) and CIA rats was detected and assessed by immunohistochemistry. **f**, **g** Immunofluorescence staining for CD68, vimentin, IGF2BP3 and DAPI in the synovium of NC and CIA rats. **h** The expression of IGF2BP3, iNOS and NLRP3 in the synovium from NC and CIA rats was assessed by western blotting. *P < 0.05, **P < 0.01, ***P < 0.001

To investigate the regulatory role of m^6^A modification in RA, the m^6^A level of peripheral blood mononuclear cells (PBMCs) was examined, which revealed that the PBMCs from RA patients presented increased m^6^A levels (Fig. S1a, 1b). Immunohistochemistry (Fig. S1c) and dot blot assays (Fig. S1d) showed that the synovial tissue of RA patients also exhibited higher m^6^A levels. In addition, the similar results were detected in the synovial tissue of CIA rats (Fig. S1e-f). Given the higher m^6^A level in RA, we analyzed the diagnostic value and biological role of 19 m^6^A regulators in RA, which found that IGF2BP3 not only has important diagnostic significance for RA, but is also closely related to cell proliferation and M1 macrophage polarization.^20^ Therefore, we focused on the mechanism by which IGF2BP3 affected the progression of RA. We detected markedly increased IGF2BP3 expression in RA synovial tissue (Fig. 1a). In addition, increased colocalization of IGF2BP3 with CD68 (a macrophage marker) was observed, which suggested increased macrophage infiltration was accompanied by increased IGF2BP3 expression in RA synovial tissue (Fig. 1b); and synovial cell (Vimentin was used as a marker of synovial cells) proliferation in RA synovial tissue was accompanied by increased IGF2BP3 expression (Fig. 1c). Western blot also indicated that IGF2BP3 and inflammatory markers (NLRP3 and iNOS) were more abundantly expressed in RA synovial tissues than in OA samples (Fig. 1d). In addition, IGF2BP3 expression was also increased in the synovial tissue of CIA rats (Fig. 1e). More importantly, the upregulation of IGF2BP3 was also closely associated with macrophage infiltration and synovial cell proliferation in the synovial tissue of CIA rats (Fig. 1f, g). Western blot showed that the occurrence of RA was accompanied by the increased expression of IGF2BP3 and inflammatory markers (NLRP3 and iNOS) (Fig. 1h).

To further explore the importance of IGF2BP3 in RA, the GEO database was analyzed. We found the IGF2BP3 expression level in RA patients’ synovial tissue was significantly increased compared with that in the normal control tissue (health people and OA patients) in the GSE90081, GSE89408 and GSE12021 datasets (Fig. S2a). More importantly, the correlations between the expression of IGF2BP3 and RA progression markers was assessed using the GSE89408 dataset, which indicated that IGF2BP3 was positively correlated with MMP1, MMP3, SPP1, IL-1β, M1 proportion, CD80, CD86 and IL-6 (Fig. S2b–i). These results further confirm that m^6^A levels and IGF2BP3 expression are increased in RA patients and suggest that IGF2BP3 is closely related to the exacerbation of RA-FLS proliferation and M1 macrophage polarization.

### IGF2BP3 regulates cell proliferation, migration, invasion, inflammatory cytokine release and autophagy in RA-FLS

**Fig. 2:**
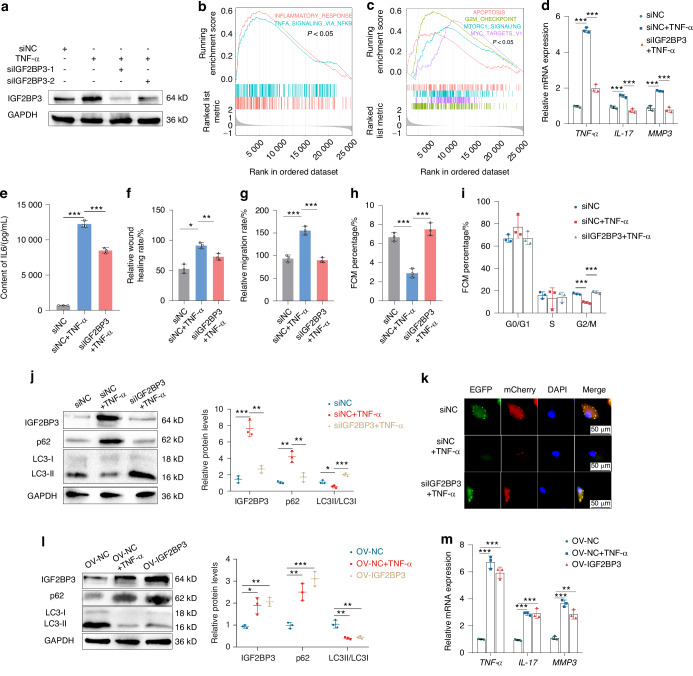
IGF2BP3 regulates cell proliferation, migration, invasion, inflammatory cytokine release and autophagy in RA-FLS. **a** The Western blot results showing IGF2BP3 expression in RA-FLS after treatment with TNF-α or siIGF2BP3. **b**, **c** GSEA revealed the enriched signaling pathways in RA patients with high IGF2BP3 expression based on hallmark gene sets. **d** The effect on TNF-α, IL-17 and MMP3 expression in RA-FLS treated with TNF-α or siIGF2BP3. **e** The content of IL-6 secreted by RA-FLS. Quantification of scratch wound healing assays (**f**) and transwell assays (**g**) of RA-FLS treated with TNF-α or siIGF2BP3. **h** RA-FLS apoptosis was measured via an Annexin V-FITC/PI staining assay. **i** Flow cytometric analysis was performed to evaluate the cell cycle distribution of RA-FLS. **j** Western blot analysis of IGF2BP3, p62 and LC3 levels in RA-FLS. **k** Representative images of the RA-FLS expressing mCherry-GFP-LC3 after treatment with TNF-α or siIGF2BP3. **l** Western blot analysis of IGF2BP3, p62 and LC3 levels in RA-FLS treated with TNF-α or overexpressing IGF2BP3. **m** The expression of TNF-α, IL-17 and MMP3 in RA-FLS treated with TNF-α or overexpressing IGF2BP3. *P < 0.05, **P < 0.01, ***P < 0.001

Given that IGF2BP3 is associated with RA-FLS proliferation, IGF2BP3 was silenced in RA-FLS. The RT_qPCR (Fig. S3a) and western blotting (Fig. S3b) indicated that the siRNA had good knockdown efficiency. To simulate the chronic inflammation and tissue destruction environment in RA, we applied 10 ng/mL TNF-α to RA-FLS.^21,22^ We found that the mRNA and protein expression levels of IGF2BP3 were significantly increased after stimulation with TNF-α, whereas siIGF2BP3 inhibited the expression level of IGF2BP3 mRNA (Fig. S3c) and protein (Fig. 2a, Fig. S3d). In addition, gene Set Enrichment Analysis (GSEA) revealed that IGF2BP3 not only is involved in the inflammatory response and TNF-α signaling, but also regulates apoptosis, G2/M checkpoint, mTORC1 signaling and MYC targets (*P* < 0.05, Fig. 2b, c). In addition, IL-6, TNF-α, IL-17 and MMP3, which are key components of phenotypic changes observed in RA lesions, were selected to evaluated the inflammatory level of RA-FLS.^23^ We found siIGF2BP3 reduced the mRNA expression levels of inflammatory cytokines, including TNF-α, IL-17 and MMP3 (Fig. 2d); and siIGF2BP3 decreased IL-6 secretion (Fig. 2e). In addition, siIGF2BP3 significantly suppressed the wound healing ability and migration ability of RA-FLS (Fig. 2f, g; Fig. S3e, f). siIGF2BP3 also inhibited the polymerization of F-actin, which participated in cell migration (Fig. S3g). Annexin V-FITC/PI staining (Fig. 2h, Fig. S3i) and TUNEL staining (Fig. S3h) indicated that siIGF2BP3 promoted cell apoptosis. The flow cytometry results also showed that siIGF2BP3 increased the proportion of G2/M-phase cells, indicating G2/M cycle arrest (Fig. 2i, Fig. S3j).

Numerous studies have shown that mTORC1 is a key regulator of autophagy, regulating different steps in the autophagy process (such as nucleation, autophagy elongation, autophagy maturation and termination).^24^ Given the GSEA results, we hypothesized that IGF2BP3 may participate in the regulation of autophagy. Western blot results showed that siIGF2BP3 decreased the expression of p62 and increased the expression of LC3, thus promoting autophagy (Fig. 2j). In addition, after the cells were infected with the mCherry-GFP-LC3 adenovirus, confocal microscopy was performed and showed that siIGF2BP3 significantly increased autophagy (Fig. 2k). In contrast, the overexpression of IGF2BP3 inhibited autophagy (Fig. 2l) and increased the mRNA expression of IL-17, MMP3, and TNF-α (Fig. 2m). Taken together, these data demonstrate that in RA-FLS, IGF2BP3 plays an important part in promoting proliferation, migration, invasion, inflammatory cytokine release and in inhibiting autophagy.

### IGF2BP3 is involved in M1 macrophage polarization by inhibiting autophagy

**Fig. 3:**
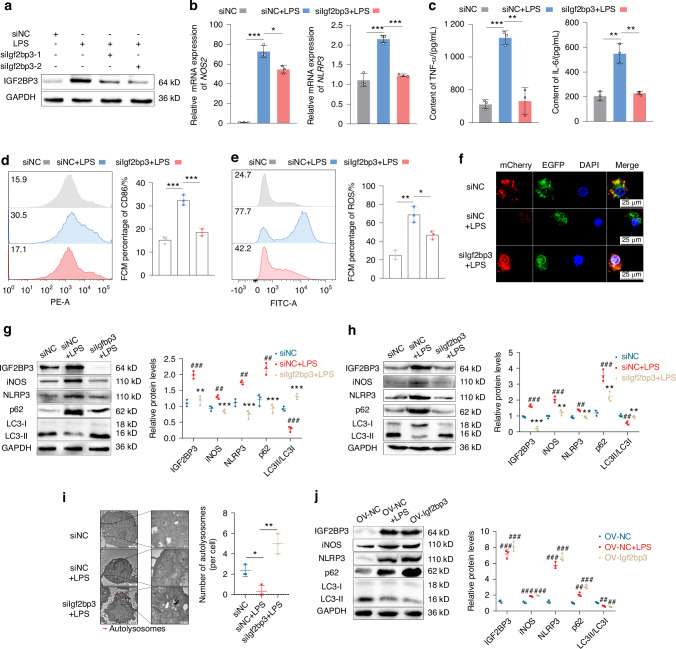
IGF2BP3 is involved in M1 macrophage polarization by inhibiting autophagy. **a** Western blot results of IGF2BP3 in RAW264.7 cells after treatment with LPS or siIGF2BP3. **b** The mRNA expression levels of NOS2 and NLRP3 in RAW264.7 cells. **c** The content of TNF-α and IL-6 secreted by RAW264.7 cells treated with LPS or siIGF2BP3. The proportions of CD86 (**d**) and ROS (**e**) in RAW264.7 cells after treatment with LPS or siIGF2BP3. **f** Representative images of RAW264.7 cells expressing mCherry-GFP-LC3.Western blot analysis showing the levels of NLRP3, iNOS, IGF2BP3, p62 and LC3 in RAW264.7 cells (**g**) and THP-1 cells (**h**) after treatment of LPS or siIGF2BP3. **i** TEM analysis of RAW264.7 cells treated with LPS or siIGF2BP3, the red arrows labelling represents autophagosomes. **j** Western blot analysis of NLRP3, iNOS, IGF2BP3, p62 and LC3 in RAW264.7 cells after treatment of LPS or IGF2BP3 overexpression. Compared with siNC/OV-NC, #*P* < 0.05, ##*P* < 0.01, ###*P* < 0.001. Compared with siNC+LPS/OV-NC + LPS, *P < 0.05, **P < 0.01, ***P < 0.001

Given that IGF2BP3 was positively correlated with M1 macrophages proportion and the expression of M1 macrophage markers (CD80 and CD86), IGF2BP3 was silenced in RAW264.7 cells. RT_qPCR and western blotting indicated that the siRNA had good knockdown efficiency (Fig. S4a, b). We further simulate the innate immune response and acute inflammation environment in RA,^25^ by applying 200 ng/mL LPS^26^ to RAW264.7 cells. We found LPS increased the mRNA (Fig. S4c) and protein (Fig. 3a, Fig. S4d) expression level of IGF2BP3, which was inhibited by siIGF2BP3. More importantly, siIGF2BP3 significantly reduced the mRNA expression levels of inflammatory cytokines, including NOS2 and NLRP3 (Fig. 3b); and siIGF2BP3 decreased TNF-α and IL-6 secretion from RAW264.7 cells (Fig. 3c). In addition, siIGF2BP3 decreased the proportion of M1 macrophages (CD86, a marker of M1 macrophages) (Fig. 3d) and ROS generation (Fig. 3e).

Increasing evidence shows that ROS are related to the inflammatory response. Overall, ROS accumulation leads to an increase in inflammatory signaling, which in turn increases ROS production. In addition, studies have shown that autophagy is a necessary pathway for reducing ROS production; defects in autophagy lead to the accumulation of ROS, which activate inflammatory signaling pathways.^27^ Given that IGF2BP3 participates in the regulation of mTORC1 signaling, we conjectured that IGF2BP3 can promote ROS production and inflammation by inhibiting autophagy. After the cells were infected with an adenovirus (mCherry-GFP-LC3), confocal microscopy revealed that the autophagic flux was significantly reduced in RAW264.7 cells stimulated with LPS, while autophagic flux was dramatically increased in IGF2BP3-silenced cells (Fig. 3f). Western blot also showed that siIGF2BP3 decreased the expression of p62 and inflammatory markers (NLRP3 and iNOS), and increased the expression of LC3 in RAW264.7 (Fig. 3g) and THP-1 cells (Fig. 3h). TEM analysis indicated LPS decreased the number of autolysosomes; whereas siIGF2BP3 increased the number of autolysosomes in RAW264.7 cells (Fig. 3i). In addition, IGF2BP3 overexpression inhibited autophagy and promoted inflammation (Fig. 3j). Together, these data demonstrate that IGF2BP3 can inhibit autophagy to increase ROS proportion, thereby increasing inflammation in macrophages.

### IGF2BP3 recognizes the m^6^A methylation site of the RASGRF1 mRNA

**Fig. 4:**
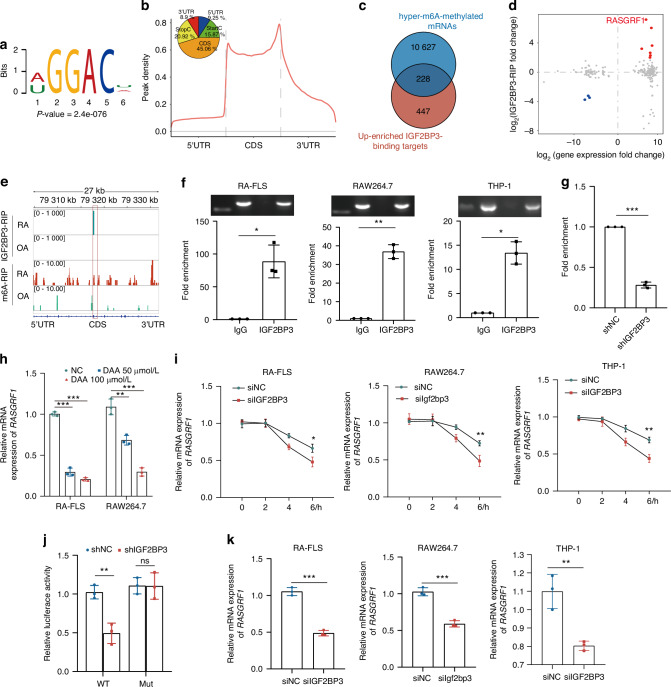
RASGRF1 is a downstream target of IGF2BP3 in m^6^A -dependent manner. **a** The consensus sequence motif identified within enriched m^6^A-binding sites. **b** Metagene profiles of the enriched m^6^A -binding sites along a normalized transcript. Pie chart depicting the fraction of significant differentially enriched m^6^A -binding sites in different transcript segments. **c** Venn diagram showing the number of overlapping up-enriched IGF2BP3 binding targets and hypermethylated mRNAs. **d** The scatter plot depicts the fold changes (log_2_) in the levels of IGF2BP3-RIP target peaks and mRNA. **e** Integrative Genomics Viewer (IGV) tracks displaying IGF2BP3 RIP-seq and MeRIP-seq read distributions along the CDS of the RASGRF1 mRNA. **f** The binding between IGF2BP3 and RASGRF1 mRNA was analyzed by RIP-qPCR. **g** shIGF2BP3 inhibited the binding between IGF2BP3 and RASGRF1 mRNA. **h** The mRNA expression levels of RASGRF1 were examined by RT_qPCR after 3-deazaadenosine (DAA) treatment. **i** RASGRF1 mRNA levels were determined by RT_qPCR in cells (siNC or siIGF2BP3) treated with actinomycin D (normalized to 0 h). **j** Relative activity of the WT or Mut luciferase reporters in shIGF2BP3-transfected THP-1 cells. **k** RASGRF1 mRNA expression levels were determined via RT-qPCR. ^ns^*P* > 0.05, *P < 0.05, **P < 0.01, ***P < 0.001

Next, we endeavored to identify the mRNAs that bind to IGF2BP3 in RA. The MeRIP-seq results revealed that in comparison with the OA group, 14 228 hypermethylated peaks and 8 639 hypomethylated peaks were present in the RA group (*P* < 0.001, |Log_2_FC | >2; Fig. S5a). An analysis of the binding sites in the RA groups indicated that ‘GGAC’ was highly concentrated at m^6^A sites as a consensus motif (Fig. 4a). Further investigation of the m^6^A peak distribution in RA groups demonstrated that most binding sites were located in the CDS region, particularly near the stop codon (Fig. 4b). To gain further insight into mRNA sites binding to IGF2BP3, we conducted IGF2BP3-RIP-seq analysis. Compared with those in the OA group, there were 335 up-enriched peaks and 866 down-enriched peaks in the RA group (*P* < 0.001, |Log_2_FC | >2; Fig. S5b). IGF2BP3 is an m^6^A RNA-binding protein whose KH domain serves as a core domain for recognizing m^6^A GC sequences, which can promote mRNA stability and the translation of downstream target genes.^28^ Therefore, to identify potential m^6^A hypermethylated targets binding to IGF2BP3 in RA, we intersected hypermethylated transcripts with up-enriched transcripts of IGF2BP3 RIP-seq, resulting in a total of 228 transcripts (Fig. 4c). Additionally, by combining transcriptome sequencing with RIP-seq and MeRIP-seq analyses, we identified seven target genes, which not only exhibited hypermethylation and high enrichment of IGF2BP3 via RIP-seq but also showed significantly increased expression (Fig. 4d, Table 1). According to previous study, RASGRF1 is a key regulatory factor in the RAS signaling pathway, which is involved in cell proliferation, inflammatory response and autophagy regulation.^29^ Therefore, we focused on RASGRF1 for further investigation. In RA, the m^6^A hypermethylated peaks and IGF2BP3 up-enriched peaks in RASGRF1 mRNA were located in CDS regions (Fig. 4e).

**Table 1:** The fold changes in the expression of genes identified via IGF2BP3-RIP_seq, RNA_seq and MeRIP_seq

Gene symbol (RA vs OA)	IGF2BP3-RIP_logFC	RNA_logFC	MeRIP-logFC
RASGRF1	6.41658517	7.181662385	4.575769816
CHST9	7.636016026	6.044717486	5.480380312
MMP3	7.802816458	3.628698561	4.026888492
GRIK2	5.445524421	3.199021619	4.903076462
COL11A1	7.373388652	2.644525165	2.568616103
ATP8A2	7.417228131	2.286430526	3.992230265
COL13A1	7.326482427	2.004165942	7.859845344

The RIP-qPCR results further revealed that IGF2BP3 bound to the RASGRF1 mRNA in RA-FLS, RAW264.7 and THP-1 cells (Fig. 4f). In addition, shIGF2BP3 inhibited the binding between IGF2BP3 and RASGRF1 mRNA in THP-1 cells (Fig. 4g). The methylation inhibitor, 3-deoxyadenosine (DAA), reduced the expression of RASGRF1 in RA-FLS and RAW264.7 cells (Fig. 4h). siIGF2BP3 also reduced the stability of RASGRF1 mRNA in RA-FLS, RAW264.7, and THP-1 cells (Fig. 4i). The online tool SRAMP (http://www.cuilab.cn/sramp)^30^ was used to predict m^6^A sites, and we constructed wild-type (WT) and mutant plasmids to examine the specific modifications of RASGRF1 (Fig. S5c). In the plasmid containing the WT RASGRF1 CDS region, shIGF2BP3 significantly decreased the expression of firefly luciferase, while plasmid containing mutated m^6^A sites did not show the effect (Fig. 4j). In addition, RT_qPCR and western blotting revealed that siIGF2BP3 significantly reduced the mRNA (Fig. 4k) and protein (Fig. S5d–f) expression levels of RASGRF1 in RA-FLS, RAW264.7 cell and THP-1cell. Taken together, these results suggest that RASGRF1 mRNA stability and expression are increased by IGF2BP3 in an m^6^A -dependent manner.

### RASGRF1 regulates RA-FLS proliferation and M1 macrophage polarization by activating mTORC1

In the GSE89408 dataset, RASGRF1 was expressed at higher levels in RA patients than in NC (health people and OA patients) (Fig. S5g). More importantly, RASGRF1 was positively correlated with MMP1, MMP3, M1 proportion, SPP1, CD80, CD86, IL-1β, and IL-6 levels (Fig. S5h). IHC staining also indicated that RASGRF1 expression was remarkably upregulated in the synovial tissue of RA patients (Fig. S6a) and CIA rats (Fig. S6b). Western blot indicated that RASGRF1 abundantly expressed in the synovial tissue of RA patients (Fig. S6c, d) and CIA rats (Fig. S6e, f). In addition, the colocalization between RASGRF1 and iNOS (a marker of M1 macrophages) was examined in synovial tissue, which revealed the upregulation of RASGRF1 in M1 macrophages from RA patients (Fig. S6g, h) and CIA synovial tissue (Fig. S6i, j).

**Fig. 5:**
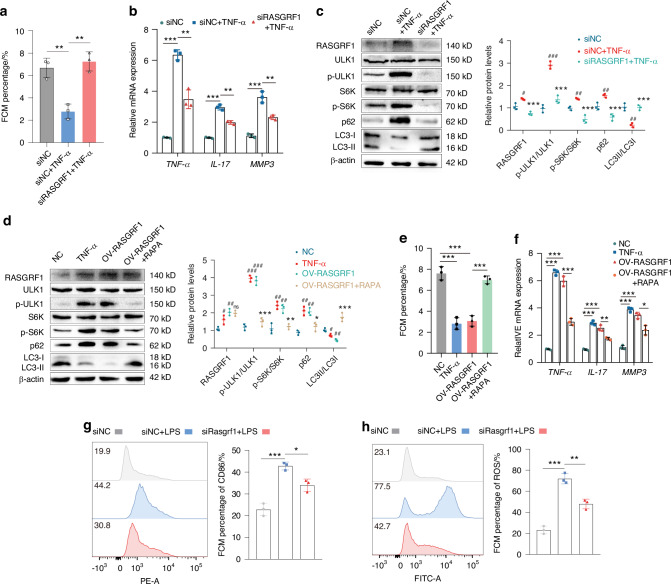
RASGRF1 regulates RA-FLS proliferation and M1 macrophage polarization by activating mTORC1. **a** The apoptosis proportion of RA-FLS after treatment of TNF-α or siRASGRF1. **b** The expression of TNF-α, IL-17 and MMP3 in RA-FLS. **c** Western blot analysis showing the levels of RASGRF1, ULK1, p-ULK1, S6K, p-S6K, p62 and LC3 in RA-FLS treated with TNF-α or siRASGRF1. **d** Western blot analysis showing the levels of RASGRF1, ULK1, p-ULK1, S6K, p-S6K, p62 and LC3 in RA-FLS treated with TNF-α, OV-RASGRF1 or RAPA. **e** The apoptosis proportion of RA-FLS. **f** The expression of TNF-α, IL-17 and MMP3 in RA-FLS. The proportion of CD86^+^ cells (**g**) and the level of ROS release (**h**) in RAW264.7 cells after treatment of LPS or siRASGRF1. Compared with siNC/NC, #*P* < 0.05, ##*P* < 0.01, ###*P* < 0.001. Compared with siNC+TNF-α/OV-RASGRF1, ^ns^*P* > 0.05, *P < 0.05, **P < 0.01, ***P < 0.001

Next, we explored the role of RASGRF1 in RA-FLS. siRASGRF1 suppressed the wound healing (Fig. S7a, c) and migration abilities (Fig. S7b, d) of RA-FLS. siRASGRF1 also inhibited the polymerization of F-actin (Fig. S7e). TUNEL staining (Fig. S7f) and Annexin V-FITC/PI staining (Fig. 5a, Fig. S7g) indicated that siRASGRF1 promoted apoptosis. Additionally, siRASGRF1 increased the proportion of G2/M-phase cells (Fig. S7h). siRASGRF1 also decreased the mRNA expression of inflammatory cytokines, including TNF-α, IL-17 and MMP3 (Fig. 5b). GSEA further revealed that RASGRF1 is not only involved in the inflammatory response and TNF-α signaling, but also regulates apoptosis, G2/M checkpoint and mTORC1 signaling, which is consistent with the GSEA results of IGF2BP3 (*P* < 0.05, Fig. S7i, j). Studies have shown that S6K and ULK1 are key downstream targets of mTORC1, and the activity of mTORC1 can be determined by detecting the phosphorylation of S6K and ULK1.^31^ Western blot analyses also indicated that siRASGRF1 significantly reduced the phosphorylation of ULK1 and S6K, and promoted autophagy (Fig. 5c). Confocal microscopy revealed that autophagic flux was increased in RASGRF1-silenced cells (Fig. S7k). To confirm whether RASGRF1 plays a functional role through mTORC1 in RA, we overexpressed RASGRF1 in RA-FLS and administered the mTORC1 inhibitor, rapamycin (MCE, Cat No. AY-22989, USA). Western blot analyses revealed that OV-RASGRF and TNF-α significantly increased the phosphorylation of ULK1 and S6K, and inhibited autophagy, which were reversed by RAPA (Fig. 5d). The proportion of apoptotic RA-FLS decreased after exposure to TNF-α or OV-RASGRF1, but was partially counteracted by RAPA. (Fig. 5e; Fig. S7l). In addition, the mRNA expression of TNF-α, IL-17 and MMP3 was significantly increased in RA-FLS stimulated with TNF-α or OV-RASGRF1, which were also reduced by RAPA (Fig. 5f). Collectively, these results indicate that RASGRF1 regulates cell proliferation, migration, invasion and inflammatory cytokine release by activating mTORC1.

Next, we investigated the role of RASGRF1 in macrophages. siRASGRF1 decreased the proportion of M1 macrophages and ROS generation (Fig. 5g, h). Given that RASGRF1 participates in the regulation of mTORC1 signaling, we speculate that RASGRF1 can also promote ROS production and activate inflammation by inhibiting autophagy. Western blot results revealed that siRASGRF1 decreased the expression of p62 and inflammatory markers (NLRP3 and iNOS), and the phosphorylation of ULK1 and S6K, and increased the expression of LC3 in RAW264.7 cells (Fig. S7m). Confocal microscopy revealed that autophagic flux was also dramatically increased in RASGRF1-silenced cells (Fig. S7n). Interestingly, overexpressing-RASGRF1 increased the phosphorylation of ULK1 and S6K, inhibited autophagy and promoted inflammation, which were also dramatically attenuated by RAPA (Fig. S7o). In addition, OV- RASGRF1 or LPS increased the proportion of M1 macrophages (Fig. S7p) and ROS generation (Fig. S7q); whereas these effects were partially counteracted by RAPA. Together, these data demonstrate that siRASGRF1 can promote autophagy to reduce ROS production by decreasing mTORC1 activation, thereby relieving inflammation in macrophages.

### RASGRF1-mediated mTORC1 activation plays a crucial role for IGF2BP3 to promote RA-FLS proliferation and inhibit autophagy

**Fig. 6:**
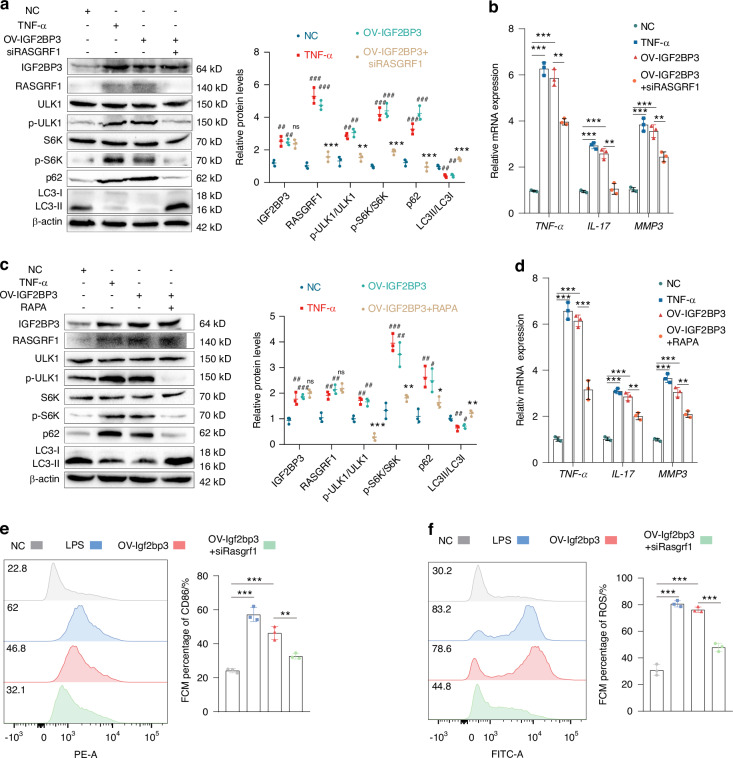
RASGRF1-mediated mTORC1 activation plays a crucial role for IGF2BP3 to promote RA-FLS proliferation and M1 macrophage polarization. **a** Western blot analysis showing the levels of IGF2BP3, RASGRF1, ULK1, p-ULK1, S6K, p-S6K, p62 and LC3 in RA-FLS treated with LPS, OV-IGF2BP3 or siRASGRF1. **b** The expression of TNF-α, IL-17 and MMP3 in RA-FLS. **c** Western blot analysis of IGF2BP3, RASGRF1, ULK1, p-ULK1, S6K, p-S6K, p62 and LC3 in RA-FLS. **d** The expression of TNF-α, IL-17 and MMP3 in RA-FLS. The proportion of CD86^+^ cells (**e**) and ROS release level (**f**) in RAW264.7 cells after the treatment of LPS, OV-IGF2BP3 or siRASGRF1. Compared with NC, #*P* < 0.05, ##*P* < 0.01, ###*P* < 0.001. Compared with OV- IGF2BP3, ^ns^ *P* > 0.05, *P < 0.05, **P < 0.01, ***P < 0.001

In order to investigate the role of RASGRRF1 in IGF2BP3-mediated biological function, we overexpressed IGF2BP3 or silenced RASGRF1 in RA-FLS. OV-IGF2BP3 promoted the wound healing ability (Fig. S8a, c) and migration ability (Fig. S8b, d) of RA-FLS. OV-IGF2BP3 also increased the polymerization of F-actin (Fig. S8e). In addition, TUNEL staining (Fig. S8f) and Annexin V-FITC/PI staining indicated that OV-IGF2BP3 inhibited cell apoptosis (Fig. S8g). OV-IGF2BP3 also decreased the proportion of G2/M-phase cells (Fig. S8h). These effects were partially counteracted by siRASGRF1. Additionally, autophagic flux was reduced in RA-FLS stimulated with OV-IGF2BP3 or TNF-α; this change was partially counteracted by siRASGRF1 (Fig. S8i). Western blot indicated that OV-IFGF2BP3 or TNF-α increased the phosphorylation of ULK1 and S6K, and inhibited autophagy, which were reversed by siRASGRF1 (Fig. 6a). What’s more, OV-IGF2BP3 significantly increased the mRNA expression of TNF-α, IL-17 and MMP3, which were also reversed by siRASGRF1 (Fig. 6b). These results indicated that RASGRRF1 plays an important role in IGF2BP3-mediated RA-FLS proliferation and inflammation.

We further confirm whether IGF2BP3 plays a functional role through mTORC1 in RA by overexpressing IGF2BP3 and administering an mTORC1 inhibitor to RA-FLS. Western blot analyses revealed that OV-IGF2BP3 or TNF-α significantly increased the phosphorylation of ULK1 and S6K, and inhibited autophagy; while, RAPA reversed these effects (Fig. 6c). The mRNA expression of TNF-α, IL-17 and MMP3 was significantly increased in RA-FLS stimulated with TNF-α or OV- IGF2BP3, which was reduced by RAPA (Fig. 6d). The reduction in apoptosis induced by TNF-α or OV-IGF2BP3 was partially counteracted by RAPA (Fig. S8j). Collectively, these results indicate that IGF2BP3 regulates RA-FLS proliferation, migration, invasion and inflammatory cytokine release by RASGRF1-mediated mTORC1 activation.

### IGF2BP3 promotes M1 macrophage polarization via RASGRF1-mediated mTORC1 activation

We further investigated the role of RASGRF1 in IGF2BP3-mediated inflammatory activation. OV-IGF2BP3 or LPS increased the proportion of M1 macrophages (Fig. 6e) and the generation of ROS (Fig. 6f), which were reversed by siRASGRF1. The reduction in autophagic flux stimulated by LPS or OV-IGF2BP3 was also partially counteracted by siRASGRF1 (Fig. S8k). Western blot indicated that OV-IGF2BP3 or LPS increased the phosphorylation of ULK1 and S6K, and the levels of inflammatory markers (NLRP3 and iNOS), and inhibited autophagy; while, siRASGRF1 reversed these changes (Fig. S8l). These results indicated that RASGRF1 plays a prominent role in IGF2BP3-mediated M1 macrophage polarization.

To further confirm whether IGF2BP3 plays a pivotal role through mTORC1 in macrophages, we overexpressed IGF2BP3 and administered an mTORC1 inhibitor to RAW264.7 cells. Western blot analyses revealed that OV-IGF2BP3 or LPS significantly increased the phosphorylation of ULK1 and S6K, and the levels of inflammation markers (NLRP3 and iNOS), and inhibited autophagy, which were counteracted by RAPA (Fig. S8m). OV-IGF2BP3 or LPS also increased the proportion of M1 macrophages (Fig. S8n) and ROS generation (Fig. S8o); while these effects were partially reversed by RAPA. Together, these results indicate that IGF2BP3 promotes M1 macrophage polarization via RASGRF1-mediated mTORC1 activation.

### IGF2BP3 knockdown alleviates RA progression

**Fig. 7:**
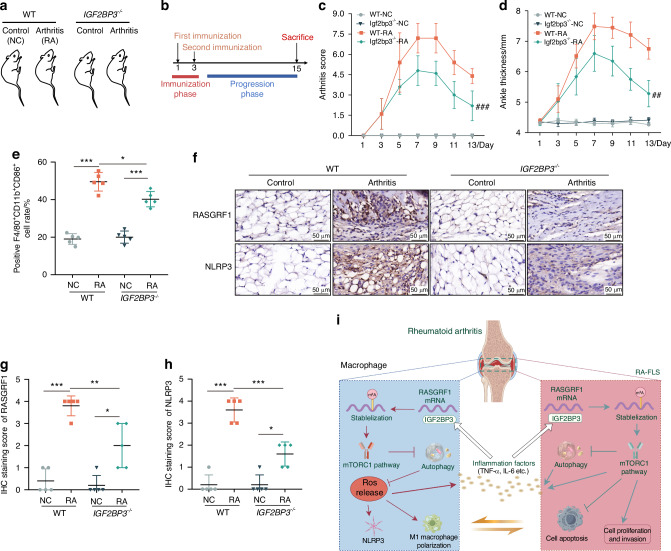
IGF2BP3 regulates the progression of arthritis in mice. **a**, **b** An illustration of the grouping and induction of mice with arthritis. **c**, **d** Effects of IGF2BP3 KO on arthritis scores and paw swelling (thickness) in mice. Compared with WT-RA, #*P* < 0.05, ##*P* < 0.01, ###*P* < 0.001. **e** The proportion of F4/80^+^CD11b^+^CD86^+^ M1 macrophages in the spleens of mice. **f**–**h** RASGRF1 and NLRP3 expression in synovium of mice with arthritis using IHC staining. **i** Schematic representation for the mechanism of IGF2BP3 to promote RA-FLS proliferation and inflammation. *P < 0.05, **P < 0.01, ***P < 0.001

To further evaluate the role of IGF2BP3 in RA, we constructed IGF2BP3 knockout (KO) mice through the deletion of two exons of IGF2BP3(exon 4 and exon 5). The knockout efficiency was confirmed by the genotyping (Fig. S9a, b). Then, we generated an arthritis model using IGF2BP3-KO mice (Fig. 7a, b). The IGF2BP3-KO arthritis mice presented decreased arthritis scores and ankle thickness than the WT arthritis mice (Fig. 7c, d). Arthrosis appearance and the micro-CT analysis also showed that the paws of the IGF2BP3-KO arthritis mice exhibited less swelling and less bone destruction than the paws of the WT arthritis mice (Fig. S9c, d). Hematoxylin-eosin (H&E) and safranin O (SO) staining also revealed a significant alleviation of synovial inflammation and hyperplasia in the IGF2BP3-KO mice compared with the WT mice (Fig. S9e, f). As expected, the levels of TNF-α and IL-6 were significantly lower in IGF2BP3-KO mice than in the WT mice (Fig. S9g, h). In addition, compared with those of WT mice with arthritis, the proportions of F4/80^+^CD11b^+^CD86^+^ M1 macrophages were lower in the spleens of IGF2BP3-KO mice with arthritis (Fig. S9i, Fig. 7e). Moreover, RASGRF1 and NLRP3 protein expression was lower in the synovial tissue of the IGF2BP3-KO arthritis mice than in that of the WT arthritis mice (Fig. 7f–h). IGF2BP3 knockdown also attenuated the up-regulation of RASGRF1 and iNOS expression in arthritic mice, which was consistent with the inflammatory score (Fig. S9j, k). Compared with those in WT arthritis mice, the expression levels of iNOS, p62 and p-S6K was lower in the synovium of IGF2BP3-KO arthritic mice, which further indicated IGF2BP3 knockdown alleviated RA progression by inhibiting mTORC1 activation and autophagy (Fig. S9l, m). In summary, these results suggest that IGF2BP3 knockdown relieved inflammatory reactions and bone destruction, and was accompanied by inhibiting RASGRF1-mediated mTORC1 activation in arthritic mice.

## Discussion

RASGRF1 is a member of the Ras-selective guanyl exchange factor family. In vitro and in vivo, RASGRF1 has exchange activity not only with H-Ras,^32^ but also with the Rho family GTPase Rac.^33^ Additionally, RASGRF1 activity can be regulated by protease-dependent post-translational modifications, and the calpain-dependent cleavage of RASGRF1 enhances its Ras-activating capacity both in vitro and in vivo.^34^ There is evidence that changes in RASGRF1 expression may lead to autoimmune diseases. In rheumatoid arthritis, RASGRF1 promotes the production of matrix metalloproteinases by regulating inflammatory processes.^35^ RASGRF1 can also activate the Rho family GTPase Rac1,^36^ and Rac1 may play a role in regulating the proliferation and invasiveness of RA-FLS by activating JNK.^37^ However, the regulatory role of RASGRF1 in RA is still unclear. In our research, we found that IGF2BP3 promotes cell proliferation and inflammatory activation by regulating RASGRF1 mRNA stability, which revealed the important regulatory role of RASGRF1 in RA progression from an epigenetic perspective.

Previous studies have reported abnormal m^6^A levels in the PBMCs of RA patients, but its role in the pathogenesis of RA remains unclear.^38^ This study showed that in RA patients, not only was the level of m^6^A in PBMC abnormal, but the level of m^6^A in synovial tissue was also significantly increased. These findings suggest that RNA methylation may participate in the progression of RA. Our previous studies revealed that IGF2BP3 was the most important regulatory factor, which may be related to synovial proliferation and the stimulation of macrophage inflammation. However, the specific mechanism of IGF2BP3 in RA has not been elucidated.^20^ In RA, RA-FLS are characterized by anchorage-independent proliferation, contact inhibition of damage, invasion and metastasis.^39^ Considering the similarity between FLS and transformed tumor cells, we examined the expression of IGF2BP3 and RASGRF1 in RA and non-RA synovial tissues. We observed that IGF2BP3 and RASGRF1 were not only highly expressed in the synovium of RA patients, but also strongly positively correlated with the production of MMP-1, MMP-3 and SPP1. In RA-FLS, IGF2BP3 regulates cell proliferation, migration, invasion, inflammatory cytokine release, and autophagy by improving RASGRF1mRNA stability. Yu Kuang et al. also reported that ALKBH5 mediates the m^6^A modification of the JARID2 mRNA and collaborates with IGF2BP3 to enhance its mRNA stability, thereby regulating the migration, invasion and proliferation of RA-FLS.^17^ In addition, we found that IGF2BP3 and RASGRF1 were closely related to M1 macrophages proportion and the levels of inflammatory markers (IL-1β, IL-6, CD80 and CD86) in the RA synovium, suggesting that IGF2BP3 and RASGRF1 are involved in the regulation of macrophage polarization. In macrophages, IGF2BP3 inhibits autophagy by increasing RASGRF1 mRNA stability, leading to ROS accumulation, thereby promoting M1 macrophage polarization and inflammasome activation. Previous studies revealed hsa_circ_0004287 reduces the stability of its host gene metastasis-associated lung adenocarcinoma transcript 1 (MALAT1) by competitively binding to IGF2BP3 with MALAT1 in an m^6^A-dependent manner. Lower levels of MALAT1 promote the ubiquitination-mediated degradation of S100A8/S100A9, thereby impeding p38/mitogen-activated protein kinase phosphorylation and macrophage-mediated inflammation.^40^ Altogether, our findings lay a strong foundation for a new mechanism by which IGF2BP3 regulates synovial cell proliferation and inflammatory activation in patients with RA.

Notably, RASGRF1-mediated mTORC1 activation plays a crucial role in the ability of IGF2BP3 to promote RA-FLS proliferation and M1 macrophage polarization. mTORC1 is a major growth regulator that senses and binds to various nutritional and environmental factors, including growth factors, energy levels, cellular stress, and amino acids; it interacts with these signals to enhance anabolism (e.g., mRNA translation and lipid synthesis) or restrict catabolism (e.g., autophagy) by phosphorylating substrates, further promoting cell growth.^41^ mTOR, which plays a crucial regulatory role in motor, metabolic, neurological, inflammatory and other diseases, has been recognized as a new target for the treatment of tumours and autoimmune diseases.^42^ Wen et al. reported that NMT1 loss causes robust synovial tissue inflammation. NMT1 has tissue-protective functions by facilitating the lysosomal recruitment of AMPK and dampening mTORC1 signaling.^43^ In addition, the inhibition of mTORC1 by ras homologue enriched in brain (Rheb1) disruption specifically in the myeloid lineage, reduces FABP4 expression in macrophages to attenuate RA development in mice.^44^ Studies have shown that S6K and ULK1 are key downstream targets of mTORC1, and mTORC1 activity can be evaluated by detecting the phosphorylation of S6K and ULK1.^31^ mTORC1 inhibits the autophagy-promoting kinase activity of the ULK1 complex by mediating the phosphorylation of specific site in ULK1 (Ser637 and Ser757) and Atg13 (Ser258). In addition, mTORC1 regulates cell growth and proliferation by mediating S6K phosphorylation, thereby regulating protein synthesis and ribosome biogenesis.^45^ In synovial cells, IGF2BP3 promotes the phosphorylation of S6K and ULK1 by activating mTORC1, enhancing the proliferative capacity of synovial cells and inhibiting autophagy. In macrophages, IGF2BP3 promotes ULK1 phosphorylation by activating mTORC1, inhibits autophagy, and leads to ROS accumulation, thereby promoting M1 macrophage polarization and inflammasome activation. And, in the K/BxN arthritis model, the *IGF2BP3*^*−/−*^ mice exhibited a milder inflammatory state and bone damage. In summary, our study broadens the current understanding of the regulatory role of RNA methylation in RA. However, small molecules targeting IGF2BP3 should be identified to improve the clinical significance of this study.

Taken together, our findings show that IGF2BP3 is highly expressed in the RA synovium, which is closely related to the exacerbation of RA-related joint injury and inflammation. IGF2BP3 improves the stability of the RASGRF1 mRNA to activate the RAS pathway, which activates mTORC1 to promote synovial cell proliferation and inflammatory activation, thereby aggravating joint injury in RA (Fig. 7i). Our findings suggest that IGF2BP3 may be a new potential target for therapeutic intervention in RA, which may provide a promising therapeutic approach for diseases associated with abnormal fibroblast activation and inflammation.

## Materials and Methods

### Cell culture and transfection

FLS were isolated from RA patients’ synovial tissue as previously described.^46^ RAW264.7 and THP-1 cells were obtained from the Chinese Academy of Sciences (Shanghai, China). All experiments used RA-FLS between passages 4 and 10. Autophagic flux was assessed using an adenovirus (mCherry-GFP-LC3, Hanheng, China).^47^

To silence the expression of IGF2BP3 and RASGRF1, IGF2BP3 siRNA (siIGF2BP3), RASGRF1 siRNA (siRASGRF1), and control siRNA (siNC) were chemically synthesized by GenePharma Co., Ltd (Shanghai, China) and transfected into cells. The coding sequences of IGF2BP3 and RASGRF1 were cloned and inserted into the PCDH-CMV lentiviral vector designed by Tsingke Biotechnology Co., Ltd. An empty lentiviral vector served as a negative control. Cell transfection was performed using Lipofectamine 3000 transfection reagent (Thermo Fisher Scientific Co. Ltd, USA).

### Human samples

The synovial tissues of RA patients, OA patients and normal controls were obtained from China-Japan Friendship Hospital. The ethics committee of China-Japan Friendship Hospital approved this study (approval number 2021-153-K111).

### Animal experiments

We constructed a CIA rat model and a K/BxN arthritis mouse model.

The CIA rat model was constructed as described in a previous study.^18^ There are two groups, including of the NC group and the CIA group. Each group included five rats.

*IGF2BP3*^*−/−*^ mice were purchased from Cyagen Biosciences. The mice used in these experiments all had a C57BL/6 background and were aged 6-8 weeks. According to previous studies, serum from adult arthritic K/BxN mice was used to construct a K/BxN arthritis mouse model via serum transfer.^48^ On day 1, the recipient mice were intraperitoneally injected with 200 µL of K/BxN mouse serum; on day 3, 200 µL serum was injected again. As previously mentioned, the clinical arthritis scores^49^ were assessed after serotransfer. On the 15th day, the mice were euthanized and histopathological changes in their feet were analyzed. Four groups were established, including WT-NC, WT- Arthritis, IGF2BP3^−/−^- NC and IGF2BP3^−/−^- Arthritis. Each group included five mice.

### Real-time quantitative PCR (RT_qPCR)

Total mRNA was extracted using a FastPure Cell/Tissue Total RNA Isolation Kit V2 (RC112-01, Vazyme, China). Sample processing and data analysis were performed as previously described in ref. ^50^ Glyceraldehyde-3-phosphate dehydrogenase (GAPDH) and β-actin were used as internal controls. Table S1 lists the primers used in this study.

### Western blot

For western blot analysis, sample processing and data analysis were performed as previously described in refs. ^50,51^ The primary antibodies used were as follows: p62 (1:1 000 dilution; Proteintech, 18420-1-AP), LC3 (1:1 000 dilution; Proteintech, 14600-1-AP), S6K(1:1 000 dilution; Proteintech, 14485-1-AP), p-S6K(1:1 000 dilution, CST, #9234), ULK1(1:1 000 dilution; Proteintech, 27352-1-AP), p-ULK1(1:1 000 dilution, CST, #5869), IGF2BP3 (1:1 000 dilution; Proteintech, 14642-1-AP), RASGRF1(1:700 dilution, Proteintech, 12958-1-AP; 1:100 dilution, Santa Cruz, sc-377234), NLRP3(1:1 000 dilution; Proteintech, 68102-1-lg), iNOS (1:1 000 dilution; Proteintech, 18985-1-AP), β-actin (1:20 000 dilution; Proteintech, 66009-1-lg) and GAPDH (1:50 000 dilution; Proteintech, 60004-1-lg) antibody.

### Enzyme-linked immunosorbent assay (ELISA)

Serum and cell supernatants were collected. According to the ELISA Kit (R&D Systems) protocol, we determine the levels of TNF-α and IL-6 in samples. Data analysis was performed as previously described in ref. ^20^

### Flow cytometry

A reactive oxygen species (ROS) detection kit (S0033S, Beyotime, China) was used to determine the amount of ROS. CD45 (BioLegend, USA), CD86 (BioLegend, USA) and CD11b (BioLegend, USA) antibodies were used to determine the proportion of M1 macrophages. Sample processing and data analysis were performed as previously described in ref. ^20^

### Immunohistochemistry

The synovial tissues of RA patients, OA patients and healthy controls were obtained from China-Japan Friendship Hospital. Sample processing and data analysis were performed as previously described in ref. ^20^ The ethics committee of China-Japan Friendship Hospital approved this study (approval number 2021-153-K111).

### RNA stability assay

To determine mRNA stability, cells transfected with siIGF2BP3 and siNC were treated with 5 μg/mL actinomycin D (Selleck, Texas, USA) for 0, 2, 4 or 6 h. The cells were collected and RNA was extracted for reverse transcription. The mRNA levels were measured by RT_qPCR.

### RNA immunoprecipitation (RIP)

RIP was performed using a Magna RIP kit (Millipore, New Bedford, MA) in accordance with the manufacturer’s protocol. Cells were lysed with RIP lysis buffer. RNA bound protein was immunoprecipitated with an IGF2BP3 antibody (Proteintech, China) and normal rabbit IgG. The coprecipitated RNA was purified and dissolved in RNase-free water. The RNA binding targets were analyzed by RT_qPCR.

### Total RNA m^6^A quantification

RNA was extracted from cells. The overall level of m^6^A modification in the cells was measured using an m^6^A methylation quantitation kit (EpiQuik). In addition, RNA m^6^A levels were quantitatively analyzed using LC-MS/MS as previously described in ref. ^52^ m^6^A dot blots were performed according to a published protocol,^53^ which was used to assess the overall level of m^6^A modification.

### MeRIP‑seq and IGF2BP3-RIP-seq

Two synovial tissue samples from RA patients and two synovial tissue samples from OA patients were used for MeRIP‑seq and IGF2BP3-RIP-seq. For MeRIP-seq, total RNA was extracted from cells using RNAiso plus (Takara) according to the manufacturer’s protocol, which was prepared for NGS. Paired-end reads were obtained from an Illumina HiSeq 6000 sequencer, and quality control was performed via Q30 calculations. After adapter trimming and low-quality read elimination with Cutadapt software (v1.9.3),^54^ all clean reads were mapped to the human reference genome with HISAT2 software (v2.0.4).^55^ Methylated sites on peaks were identified with MACS2 peak-calling software (v2.1.1),^56^ where the corresponding input sample served as a control. Differentially methylated sites were identified with diffReps (v1.55.6).^57^ Peaks identified by overlapping mRNA exons were determined and chosen with our original scripts. Motifs enriched with m^6^A peaks were identified with DREME (v5.4.1).^58^ m^6^A peak distributions were visualized with the Integrative Genomics Viewer (IGV).

RIP was performed using a Magna RIP Kit (Millipore) according to the manufacturer’s instructions. The input and immunoprecipitated RNAs were recovered and subjected to next-generation sequencing (NGS) or RT_qPCR analysis. Both the input and immunoprecipitated RNA samples were subjected to quality-controlled and used to generate RNA-seq libraries using the GenSeq® Low Input RNA Library Prep Kit. Sequencing was performed with an Illumina NovaSeq platform in paired-end-read mode, with 150 bp per read. The sequencing reads were aligned to the human genome with HISAT2 (v2.0.4). RIP peaks were calculated with diffReps (v1.55.6). Peaks were annotated with the annotatePeaks.pl module of HOMER (v4.9.1), using the default settings.^59^ Motifs were identified with the findMotifsGenome.pl module.^60^ A metagene analysis was performed to map the mRNA peak distribution using the Guitar Bioconductor package (v1.20.1).^61^

### Statistical analysis

Each experiment was independently repeated three times, and the data are presented as the means ± standard errors (SEMs), unless indicated otherwise. R version 4.0.4 software (Institute for Statistics and Mathematics, Vienna, Austria; https://www.r-project.org) and GraphPad Prism software (GraphPad Software, San Diego, CA, USA) were used for statistical analyses and graphing. *P* < 0.05 was considered statistically significant.

## Supplementary information

SUPPLEMENTAL MATERIAL

## Data Availability

The data of MeRIP_seq and IGF2BP3-RIP_seq have been deposited at GEO database: GSE273245 and GSE273246. The data are available upon reasonable request.
