# Supplementary material for: IGF2BP3-mediated m6A modification of RASGRF1 promoting joint injury in rheumatoid arthritis
Source: Bone Res. 2025 May 12;13:51. doi: 10.1038/s41413-025-00434-z (PMC12069659; doi:10.1038/s41413-025-00434-z)
Supplement: Supplementary file 1 — SUPPLEMENTAL MATERIAL [file 41413_2025_434_MOESM1_ESM.docx]

**Supplementary material**

Supplementary Table 1 The sequences of the primers used for RT_qPCR.

| **Gene** | **Forward sequence (5′-3′)** | **Reverse sequence (5′-3′)** |
| --- | --- | --- |
| Human |  |  |
| TNF-α | GAGGCCAAGCCCTGGTATG | CGGGCCGATTGATCTCAGC |
| IL-17 | TCCCACGAAATCCAGGATGC | GGATGTTCAGGTTGACCATCAC |
| MMP3 | AGGCAAGACAGCAAGGCATA | ACGCACAGCAACAGTAGGAT |
| RASGRF1 | TCCTGTCGTGAACTGGACAAT | CTGCACTGGCTAAGGACATCC |
| IGF2BP3 | ACGAAATATCCCGCCTCATTTAC | GCAGTTTCCGAGTCAGTGTTCA |
| β-actin | CCTTCCTGGGCATGGAGTC | TGATCTTCATTGTGCTGGGTG |
| Mouse |  |  |
| RASGRF1 | GCCAGAAGACTTGACAACGCT | TCAATCTACAGGGATGGTGGAAG |
| IGF2BP3 | CCTGGTGAAGACGGGCTAC | TCAACTTCCATCGGTTTCCCA |
| NLRP3 | ATTACCCGCCCGAGAAAGG | TCGCAGCAAAGATCCACACAG |
| NOS2 | GTTCTCAGCCCAACAATACAAGA | GTGGACGGGTCGATGTCAC |
| Gapdh | AAATGGTGAAGGTCGGTGTGAAC | CAACAATCTCCACTTTGCCACTG |


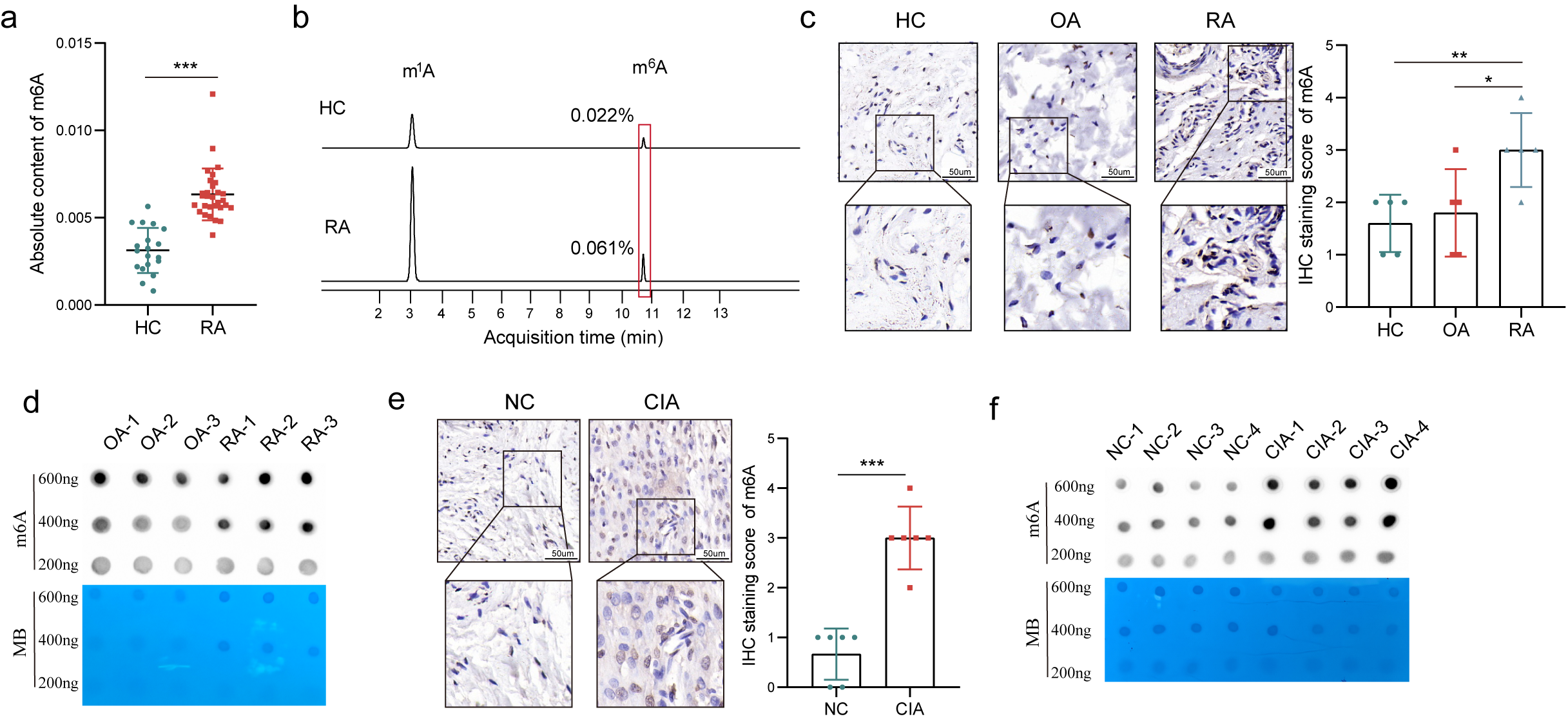


Supplementary Fig. 1 The level of m^6^A and IGF2BP3 is upregulated in RA patients. (a)The absolute content of m^6^A in PBMCs of HCs and RA patients. (b) Representative peak map of the absolute content of m^6^A detected using the LC‒MS/MS assay in HCs and RA patients. The level of m^6^A in synovium from HC, OA and RA was detected by immunohistochemistry (c) and dot blot assays (d). The level of m^6^A in the synovium from NC and CIA rats was assessed by immunohistochemistry (e) and dot blot assays (f). MB, methylene blue. *p<0.05, **p<0.01, ***p<0.001.


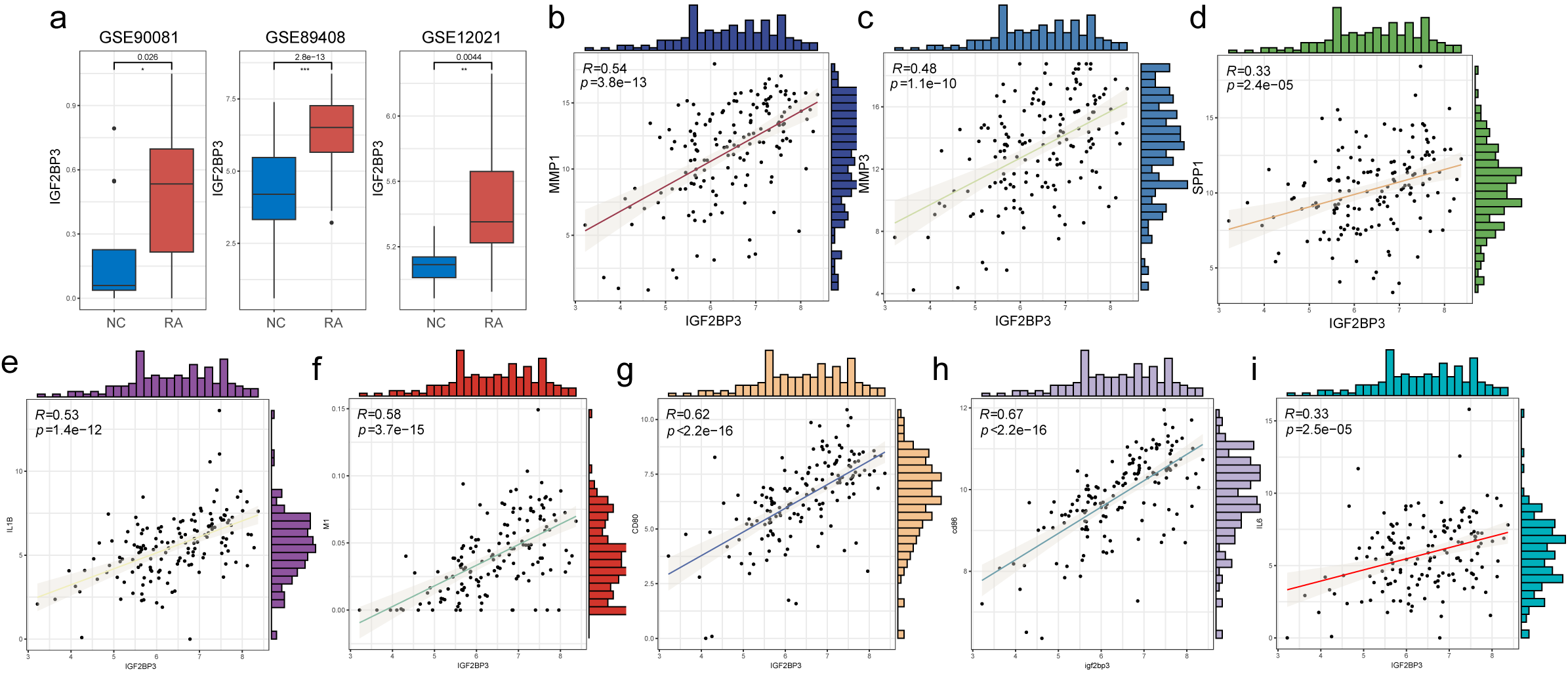


Supplementary Fig. 2 IGF2BP3 expression in synovium is increased and correlated with inflammatory markers in RA. (a) IGF2BP3 expression level of the RA and NC groups in the GSE90081, GSE89408 and GSE12021 datasets. (b-i) Correlations between MMP1, MMP3, SPP1, IL1β, M1 proportion, CD80, CD86, IL6 and the expression level of IGF2BP3 in RA patients.

**
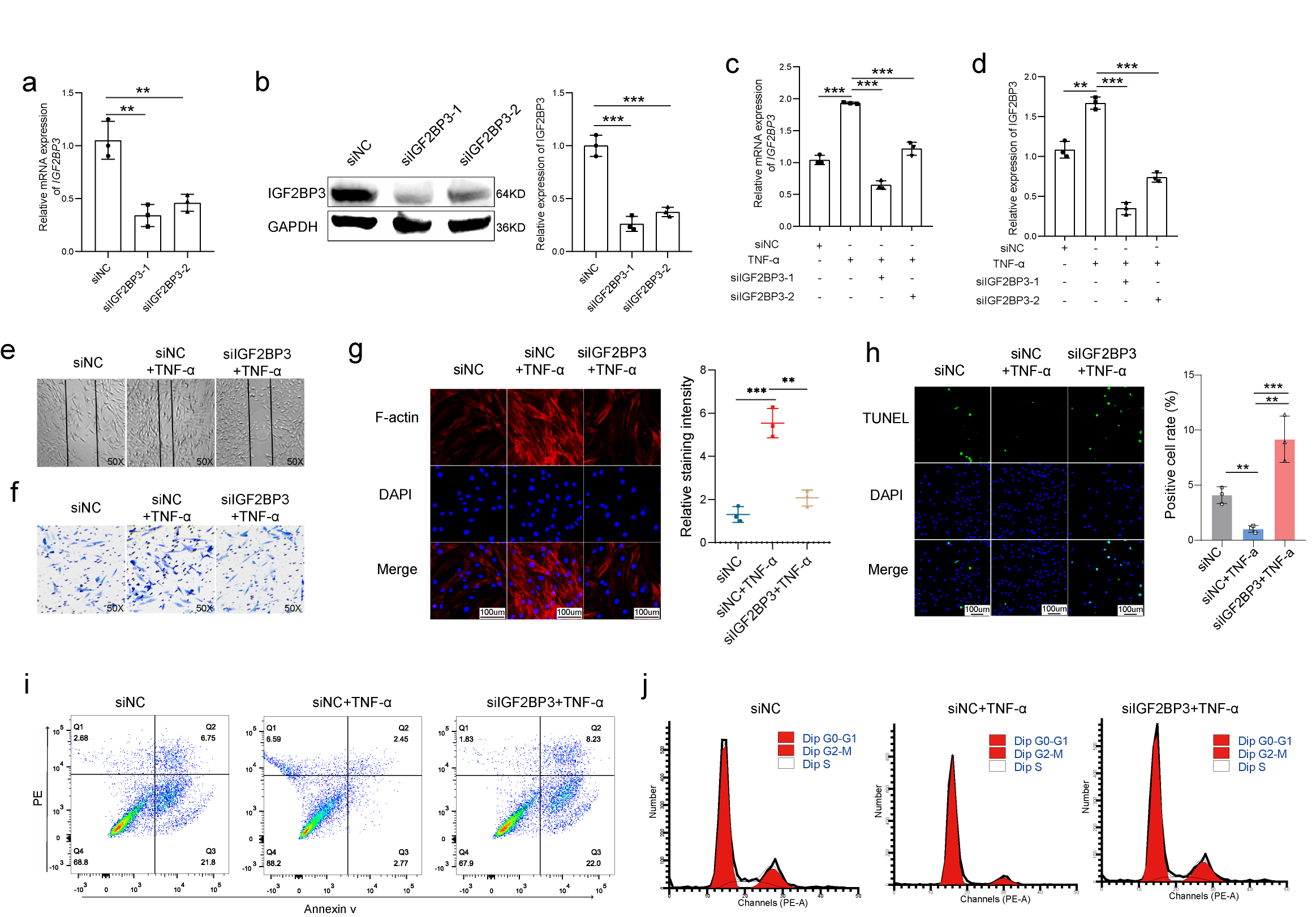
**

Supplementary Fig. 3 IGF2BP3 regulates cell proliferation, migration and invasion. The RT_qPCR (a) and Western blot (b) results of IGF2BP3 in RA-FLS after siIGF2BP3 transfection. The RT_qPCR (c) and Western blot (d) analysis of IGF2BP3 expression in RA-FLS after treatment with TNF-α or siIGF2BP3. Representative images of scratch assays (e) and transwell assays (f) of RA-FLS treated with TNF-α or siIGF2BP3. (g) The F-actin expression in RA-FLS after treatment of TNF-α or siIGF2BP3. (h) TUNEL (green) staining of RA-FLS treated with TNF-α or siIGF2BP3. Representative images of cell apoptosis (i) and cell cycle distribution (j) of RA-FLS determined by Flow cytometric. *p<0.05, **p<0.01, ***p<0.001.


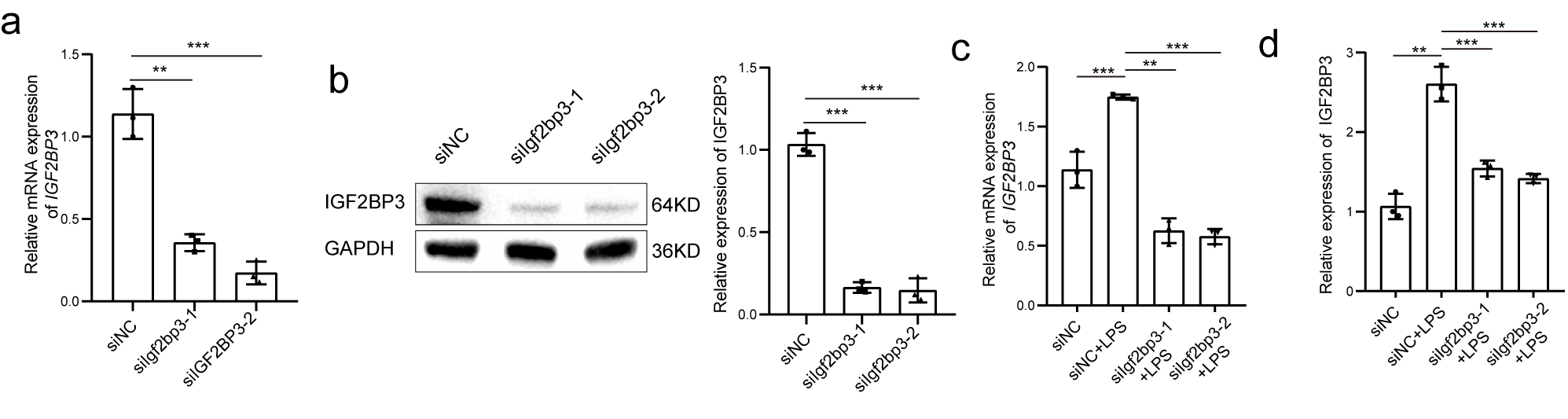


Supplementary Fig. 4 The knockdown efficiency of siIGF2BP3 in RAW264.7 cells. RT_qPCR (a) and Western blot (b) results of IGF2BP3 in RAW264.7 cells after siIGF2BP3 transfection. RT_qPCR (c) and western blot (d) results of IGF2BP3 in RAW264.7 cells after treatment with LPS or siIGF2BP3. *p<0.05, **p<0.01, ***p<0.001.


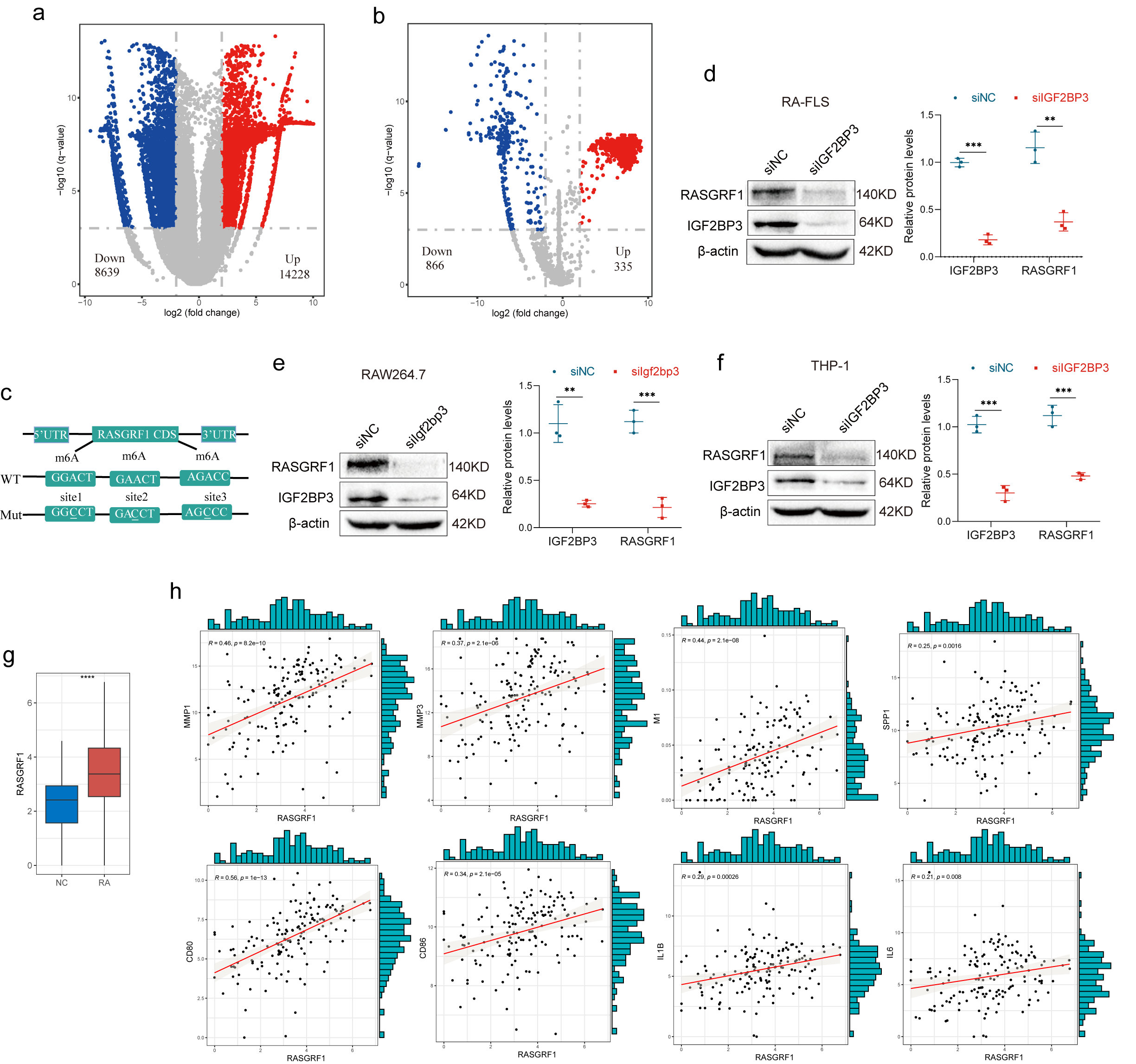


Supplementary Fig. 5 RASGRF1 mRNA is the target of IGF2BP3 in RA. (a) The volcano plot of altered m^6^A peaks identified by MeRIP-seq in OA and RA groups. (b) The volcano plot of changed IGF2BP3-RIP target peaks identified by IGF2BP3-RIP-seq in OA and RA groups. (c) Putative m^6^A modification sites in the CDS of RASGRF1 and synonymous mutations in the RASGRF1 CDS. RASGRF1 protein (d-f) expression levels were determined via Western blot in RA-FLS, RAW264.7, and THP-1 cells treated with siNC or siIGF2BP3. (g) RASGRF1 expression level of the RA and NC groups in the GSE89408 dataset. (h) Correlations between MMP1, MMP3, SPP1, IL1β, M1 proportion, CD80, CD86, IL6 and the expression level of RASGRF1 in RA patients. *p<0.05, **p<0.01, ***p<0.001.


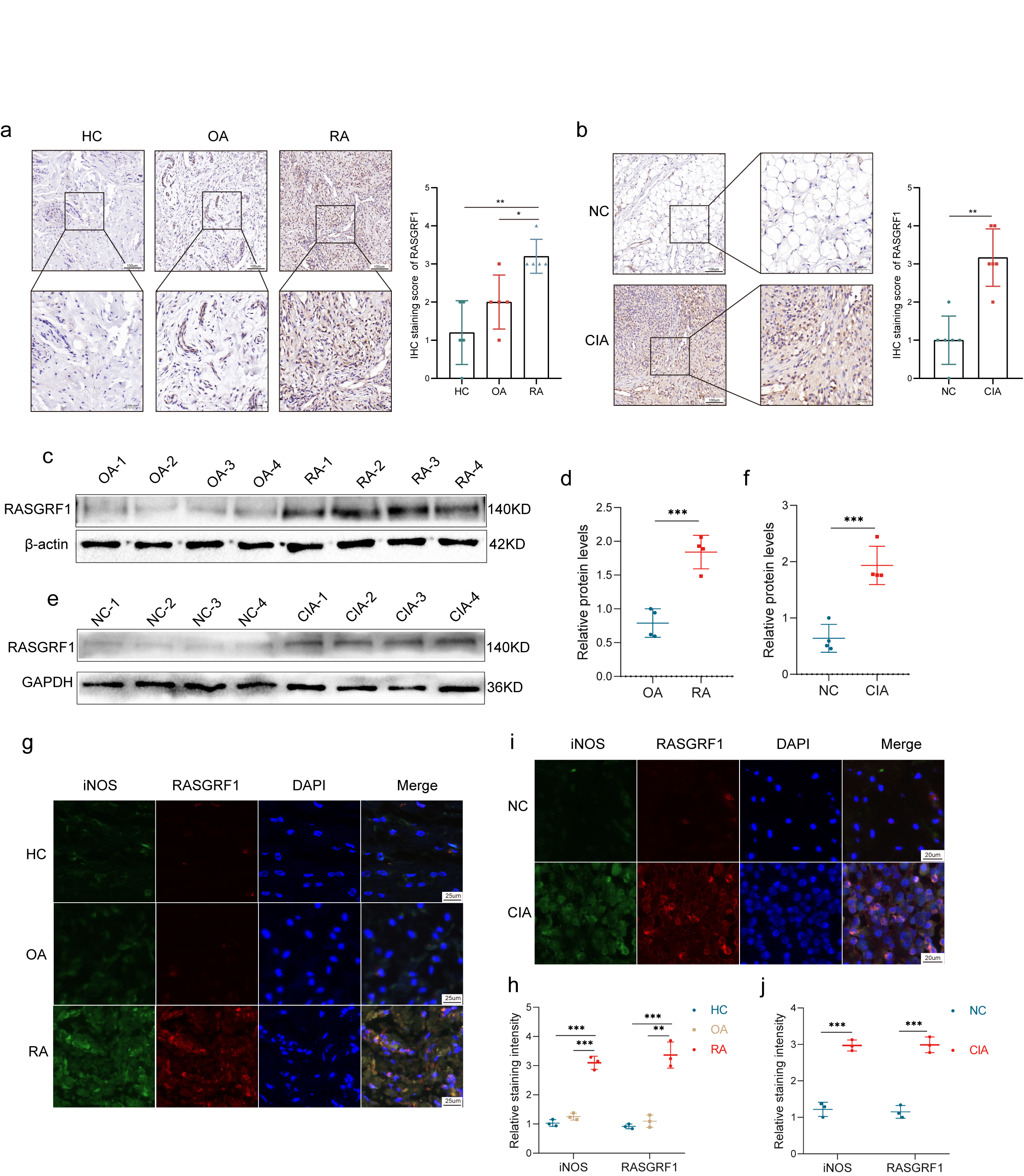


Supplementary Fig. 6 RASGRF1 expression is upregulated in RA and is related to the expression of IGF2BP3. (a) RASGRF1 expression in synovial tissue from HC, OA and RA was detected by immunohistochemistry. (b) The expression of IGF2BP3 in synovial tissue from NC and CIA rats was assessed by immunohistochemistry. (c-d) The expression level of RASGRF1 was assessed in synovium from RA and OA. (e-f) The expression of RASGRF1 was assessed in synovium from NC and CIA rats. (g-h) Immunofluorescence staining and quantitative analysis for iNOS, RASGRF1 and DAPI in synovium from HC, OA and RA. (i-j) Immunofluorescence staining and quantitative analysis for iNOS, RASGRF1 and DAPI in synovium of NC and CIA rats. *p<0.05, **p<0.01, ***p<0.001.


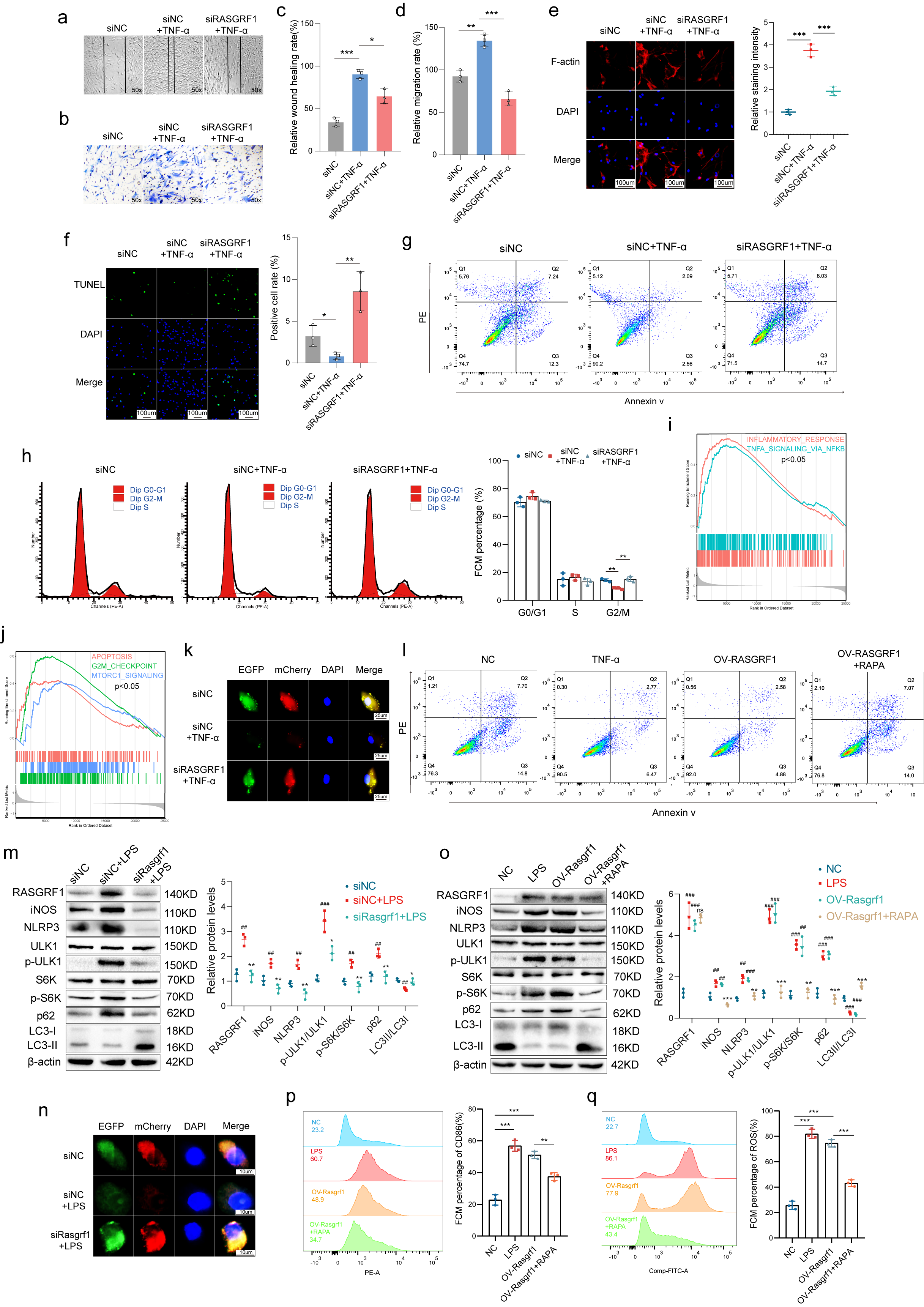


Supplementary Fig. 7 RASGRF1 is involved in RA-FLS proliferation and M1 macrophage polarization. The quantification and representative images of scratch assays (a, c) and transwell assays (b, d) of RA-FLS treated with TNF-α or siRASGRF1. (e) The F-actin expression level in RA-FLS. (f) TUNEL (green) staining of RA-FLS treated with TNF-α or siRASGRF1. The quantification and representative images of cell apoptosis (g) and cell cycle distribution (h) of RA-FLS determined after treatment of TNF-α or siRASGRF1. (i-j) GSEA revealed the enriched signaling pathways in RA with high RASGRF1 expression based on the hallmark gene sets. (k) Representative images of RA-FLS expressing mCherry-GFP-LC3 after treatment with TNF-α or siRASGRF1. (l) Representative images of cell apoptosis of RA-FLS treated with TNF-α, RAPA or overexpressing RASGRF1. (m) Western blot analysis of RASGRF1, NLRP3, iNOS, ULK1, p-ULK1, S6K, p-S6K, p62 and LC3 in RAW264.7 cells treated with LPS or siRASGRF1. Compared with siNC, #p<0.05, ##p<0.01, ###p<0.001. Compared with siNC+LPS, ^ns^p>0.05, *p<0.05, **p<0.01, ***p<0.001. (n) Representative images of RAW264.7 cells expressing mCherry-GFP-LC3 after treatment with LPS or siRASGRF1. (o) Western blot analysis of RASGRF1, NLRP3, iNOS, ULK1, p-ULK1, S6K, p-S6K, p62 and LC3 in RAW264.7 cells. Compared with NC, #p<0.05, ##p<0.01, ###p<0.001. Compared with OV-Rasgrf1, ^ns^p>0.05, *p<0.05, **p<0.01, ***p<0.001. The proportion of CD86^+^ cells (p) and ROS content (q) in RAW264.7 cells treated with LPS, overexpressing RASGRF1 or RAPA. *p<0.05, **p<0.01, ***p<0.001.


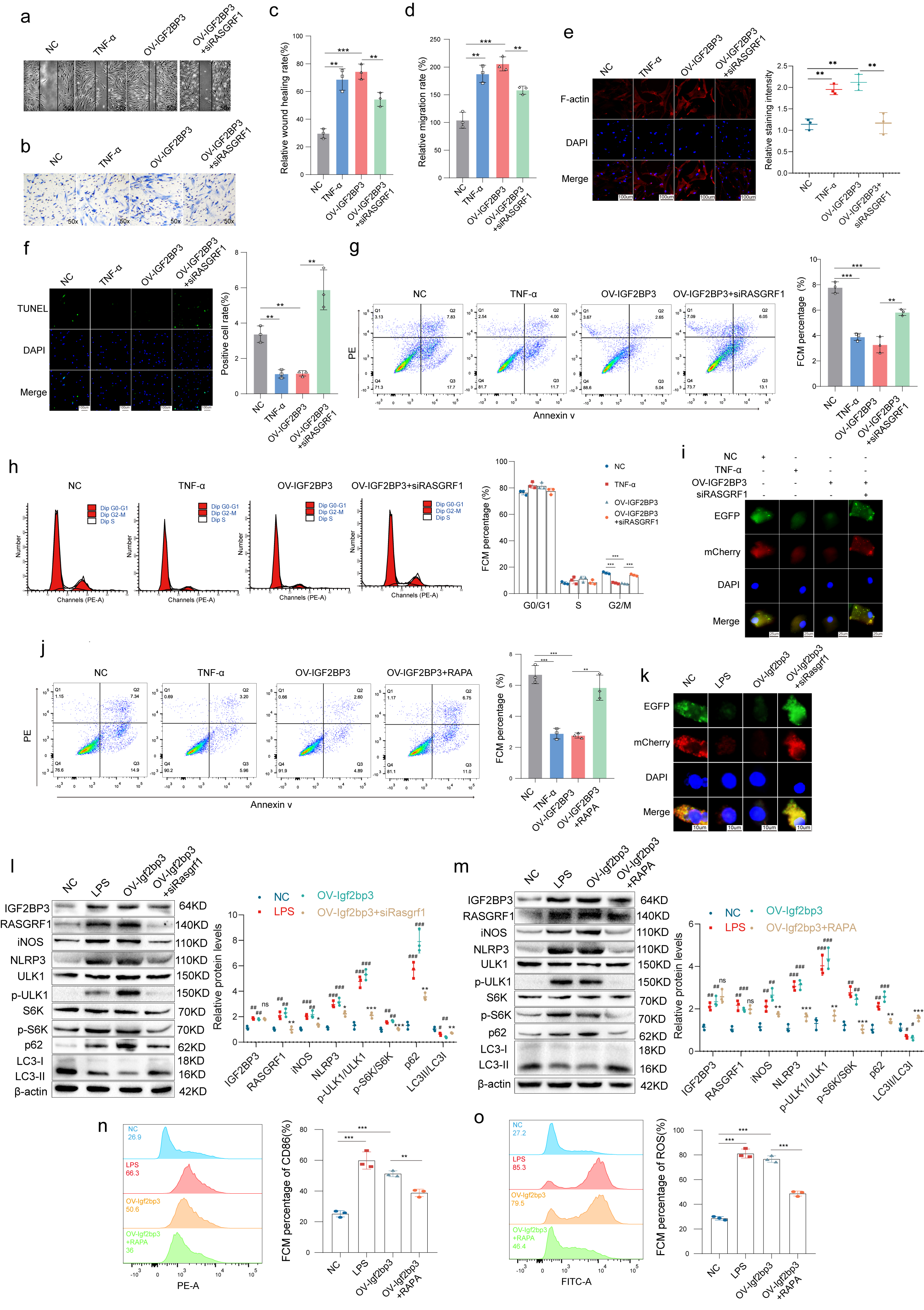


Supplementary Fig. 8 IGF2BP3 promotes RA-FLS proliferation and M1 macrophage polarization by RASGRF1-mediated mTORC1 activation. The quantification and representative images of scratch assays (a, c) and transwell assays (b, d) of RA-FLS treated with TNF-α, OV-IGF2BP3 or siRASGRF1. (e) The F-actin expression in RA-FLS. (f) TUNEL (green) staining of RA-FLS treated with TNF-α, OV-IGF2BP3 or siRASGRF1.The cell apoptosis (g) and cell cycle distribution (h) of RA-FLS determined after treatment of TNF-α, OV-IGF2BP3 or siRASGRF1. (i) Representative images of the RA-FLS expressing mCherry-GFP-LC3 after treatment of TNF-α, OV-IGF2BP3 or siRASGRF1. (j) The cell apoptosis proportion of RA-FLS treated with TNF-α, RAPA or overexpressing IGF2BP3. (k) Representative images of the RAW264.7 cells expressing mCherry-GFP-LC3 after treatment with LPS, OV-IGF2BP3 or siRASGRF1. (l) Western blot analysis of IGF2BP3, RASGRF1, NLRP3, iNOS, ULK1, p-ULK1, S6K, p-S6K, p62 and LC3 in RAW264.7 cells treated with LPS, OV-IGF2BP3 or siRASGRF1. (m) Western blot analysis of IGF2BP3, RASGRF1, NLRP3, iNOS, ULK1, p-ULK1, S6K, p-S6K, p62 and LC3 in RAW264.7 cells treated with LPS, OV-IGF2BP3 or RAPA. Compared with NC, #p<0.05, ##p<0.01, ###p<0.001. Compared with OV- Igf2bp3, ^ns^ p>0.05, *p<0.05, **p<0.01, ***p<0.001. The proportion of CD86^+^ cells (n) and ROS content (o) in RAW264.7 cells treated with LPS, overexpressing IGF2BP3 or RAPA. *p<0.05, **p<0.01, ***p<0.001.


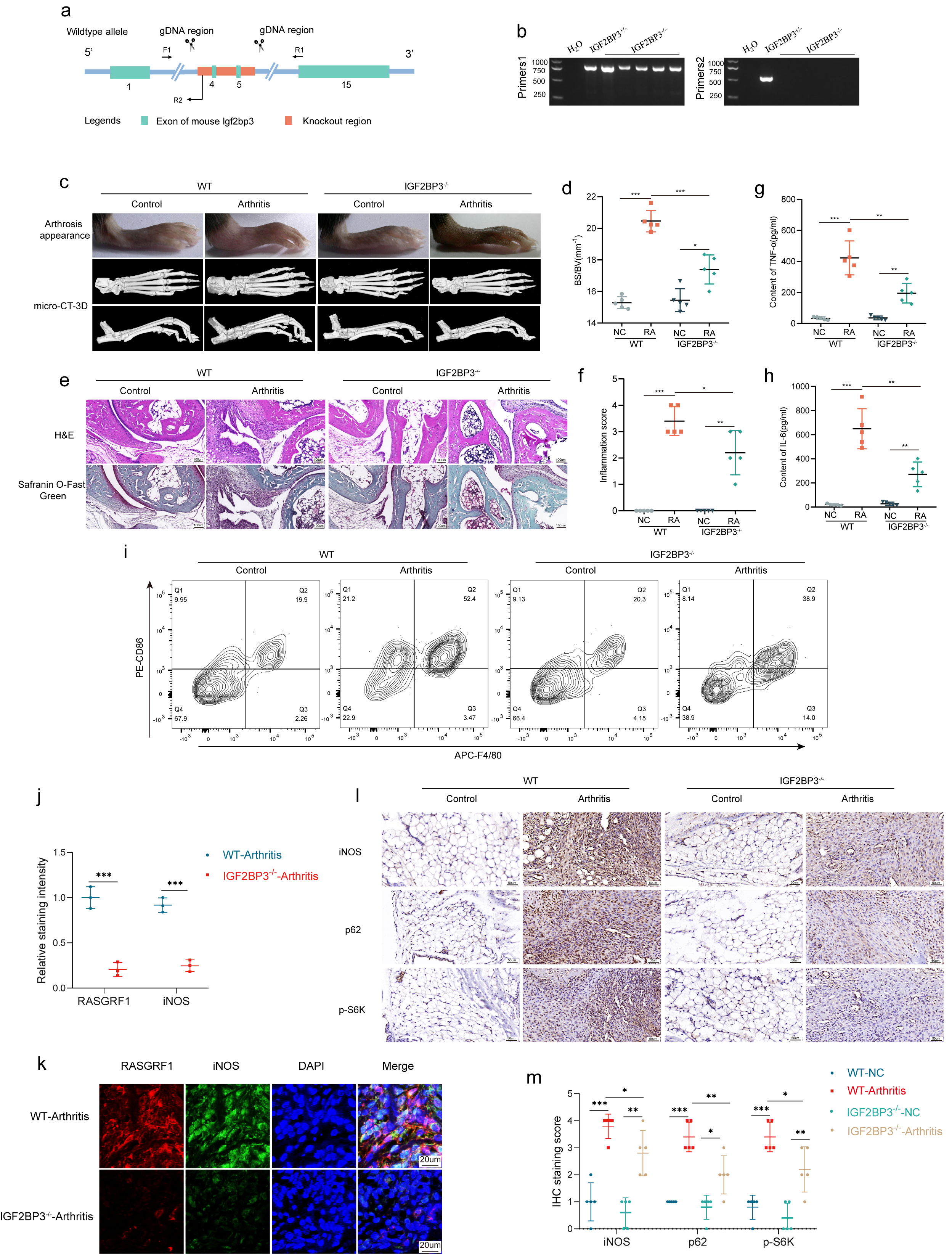


Supplementary Fig. 9 IGF2BP3 knockdown alleviates RA progression via promoting mTORC1 activation and autophagy. (a-b) Genotyping and confirmation of IGF2BP3 knockout mice. (c) Representative images of the arthrosis appearance and micro-CT scans of the mice. (d) The quantitative analysis of BS/BV of ankle. (e) Representative images of the H&E and Safranin O/Fast green staining. (f) The quantitative analysis of histological scores. Protein levels of TNF-α (g) and IL-6 (h) in the peripheral serum. (i) The proportion of F4/80^+^CD11b^+^CD86^+^ M1 macrophages in the spleens of mice. (j-k) Representative plots of RASGRF1 (red) and iNOS (green) staining in mice’ synovium. (l-m) The iNOS, p62 and p-s6k expression in synovium of mice with arthritis using IHC staining. ^ns^ p>0.05, *p<0.05, **p<0.01, ***p<0.001.
